# 肿瘤浸润性淋巴细胞在恶性血液系统疾病中的研究进展

**DOI:** 10.3760/cma.j.cn121090-20230912-00113

**Published:** 2024-06

**Authors:** 如冰 郑, 毅 肖

**Affiliations:** 华中科技大学同济医学院附属同济医院血液科，武汉 430030 Department of Hematology, Tongji Hospital, Tongji Medical College, Huazhong University of Science and Technology, Wuhan 430030, China

## Abstract

近年来，免疫疗法在肿瘤治疗中进展迅速，其中免疫活性细胞的过继免疫治疗在恶性血液系统疾病的治疗中也越来越受到重视。肿瘤浸润性淋巴细胞（tumor-infiltrating lymphocyte，TIL）是一类以T细胞为主的异型淋巴细胞，具有高度异质性。作为肿瘤微环境的重要组成部分，TIL在恶性肿瘤的发生发展中至关重要。TIL是继淋巴因子激活杀伤细胞之后发现的一种新型免疫活性细胞，在不需要大量IL-2诱导的情况下即可展现出强特异性和有效性。本文就TIL的组成、特点和其在恶性血液系统疾病中的研究进展等进行综述。

## Abstract (en)

In recent years, immunotherapy has been progressing rapidly in tumor treatment, among which, adoptive immunotherapy of immunologically active cells has also gained increasing attention in the treatment of malignant hematological diseases. Tumor-infiltrating lymphocytes are a heterogeneous class of T-cell-based lymphocytes with high heterogeneity. As an important component of the tumor microenvironment, TILs are crucial in the development of malignant tumors. TILs are a new type of immunoreactive cells discovered after lymphokine-activated killer cells, which can show high specificity and efficacy without the need for large amounts of interleukin-2. Tumor immunotherapy with TILs has shown encouraging results and is valuable in determining patient prognosis. In this paper, we review the composition and characteristics of TILs and their progress in malignant hematologic diseases.

恶性血液系统疾病源于造血干细胞的异常增殖与分化，其全身侵袭性和难治性是治疗的主要挑战。尽管放疗、化疗、造血干细胞移植及靶向治疗等方法在临床上广泛应用，但其往往难以根治疾病，且有较高的复发率和不良预后。相比之下，细胞免疫治疗通过增强或恢复机体免疫系统功能，或直接靶向肿瘤细胞，展现出清除肿瘤细胞和降低不良反应的潜力。特别是肿瘤浸润性淋巴细胞（TIL）作为肿瘤微环境（TME）中的主要T细胞来源，展现出更强的增殖活性、肿瘤特异性和更轻微的不良反应，标志着其在细胞过继免疫治疗领域具有显著的应用前景[1]。

一、TIL的概述

TIL是肿瘤间质中具有异质性的一类淋巴细胞，以T细胞为主，还包括在抗肿瘤免疫中发挥巨大作用的B细胞、自然杀伤（NK）细胞和抑制肿瘤免疫反应的树突状细胞、髓系来源抑制细胞，以及在TME中可以极化为M_1_型和M_2_型的巨噬细胞等[2]。T细胞包括辅助性T（Th）细胞、细胞毒性T细胞和调节性T（Treg）细胞。大多数T细胞以CD3^＋^、CD4^+^和CD8^+^ T细胞为主，是对肿瘤起识别、抵抗和攻击作用的细胞群。TIL表型具有异质性，不同肿瘤来源的TIL中，CD4^+^ T细胞与CD8^+^ T细胞的比例存在差异，大多数情况下以CD8^+^ T细胞为主[3]。在体外应用IL-2激活TIL有不同的表型，只有将充分激活、处于效应最高的激活态细胞输给患者时疗效更好。

TIL主要通过细胞免疫反应来发挥抗肿瘤效应：释放细胞毒素直接杀伤肿瘤细胞，增强机体免疫从而提高机体对肿瘤细胞的杀伤作用。研究结果显示[4]，在TME中CD8^+^ T细胞的大量浸润通常与肿瘤预后较好相关，因其具有直接杀伤肿瘤细胞的能力，如卵巢肿瘤和结直肠肿瘤。相反，免疫抑制细胞的浸润与肿瘤预后较差相关，因其限制了机体对肿瘤的免疫反应。因此，TIL的数量和质量是抗肿瘤免疫反应结果的关键决定因素。TIL还能增强其他免疫疗法的疗效：由于TIL中的CD8^+^ T细胞能介导γ干扰素（IFN-γ）的表达和分泌，而IFN-γ分泌增多又会诱导肿瘤细胞内的程序性死亡受体配体-1（PD-1）的表达和上膜[5]，故TIL联合抗PD-1药物治疗可提高其治疗效果。另外，TIL中的T淋巴细胞群表面TCR具有多样性，能够识别多个靶点，识别一系列肿瘤抗原，可以较大程度避免肿瘤异质性免疫逃避[6]。在TME中，肿瘤细胞与免疫细胞间相互作用对于控制恶性肿瘤的发展和进展至关重要。如Treg细胞、Th2细胞对肿瘤细胞具有免疫抑制作用，CD8^＋^ T细胞是抗肿瘤免疫反应的主要效应细胞。TIL对改善患者预后具有重要意义，并且在免疫治疗时代显示出预测疗效的潜力[7]。正是TIL的这些特性使其应用于血液肿瘤中时，表现出了对肿瘤细胞的强浸润性、高特异性、较低的脱靶毒性以及抗肿瘤异质性的优势。

二、TIL在血液肿瘤中的研究进展

（一）TIL与白血病

1. TIL与急性髓系白血病（AML）：在AML治疗的研究领域中，Cao等[8]从AML患者的骨髓中提取了TIL，这些TIL主要是CD3^+^ T细胞。经过体外扩增后，这些CD3^+^ T细胞可以对体外AML原始细胞发挥细胞毒性作用，并能够浸润到骨髓中并留存于移植到免疫缺陷小鼠体内的预注射自体AML原始细胞周围。经过基因工程改造后，这些TIL还可以表达CYP27B1基因，这一创新不仅提供了研究CYP27B1^+^ TIL对抗白血病功能的新途径，也暗示了TIL作为未来基因疗法潜在的细胞载体的可能性。在预测AML患者的复发率和总生存（OS）期方面，Le等[9]发现淋巴细胞绝对计数（ALC）是一个评价AML患者预后的独立预测指标。当机体免疫耐受减弱而肿瘤抗原性增强时，淋巴细胞的作用与TIL类似，可能代表了机体免疫系统对肿瘤细胞的反应，这是免疫系统对肿瘤细胞稳定发挥作用的标志。

2. TIL与急性淋巴细胞白血病（ALL）：TIL对ALL不仅具有判断预后的作用，而且能发挥抗肿瘤作用的免疫功能。Riva等[10]分析了10位Ph^+^ ALL患者，发现患者接受高剂量甲磺酸伊马替尼治疗后，骨髓和外周血中长时间存在TIL，并且还产生了IFN-γ、TNF-α和IL-2等细胞因子。另外值得注意的是，骨髓中TIL细胞量不仅明显比外周血增多，而且与微小残留病（MRD）呈负相关，这凸显了TIL在评估预后中的重要作用。Zamora等[11]在ALL儿童患者的单细胞水平上检测了CD8^+^ TIL的表型和转录谱，发现了包括高功能效应子在内的异质群体。进一步的研究表明，尽管ALL患者的突变负荷低，但仍诱导了强大的抗肿瘤免疫应答。

3. TIL与慢性髓性白血病（CML）：CML是一种以髓系细胞过度增殖为特征的白血病。酪氨酸激酶抑制剂（TKI）是目前患者使用的标准治疗药物，但其会带来很多不良反应。研究者们开始关注免疫细胞治疗CML，NK细胞具有细胞毒性并且能在释放各种细胞因子后产生适应性免疫反应，在CML的抗肿瘤免疫反应中起重要作用。开展NK细胞介导的免疫疗法时可以通过扩大NK细胞群和增强整体功能来提高NK细胞的活化[12]。当TKI治疗无效时，NK细胞可以发挥治疗作用。在造血干细胞移植中，NK细胞的同种异体反应性可以降低CML的复发率。另一个值得关注的问题是CML患者中的NK细胞毒性会随着白血病细胞危象的进展而下降。为解决这一问题，有研究开展了其他基于NK细胞的免疫疗法类型包括过继转移、单克隆抗体治疗和嵌合抗原受体-NK（CAR-NK）细胞治疗等[13]。这部分的深入研究对于未来开发NK细胞治疗CML具有潜在的应用前景。

4. TIL与慢性淋巴细胞白血病（CLL）：CLL是一种免疫抑制性强的疾病。CLL患者血液中的CD4^＋^ T细胞占总T细胞比例较正常人偏高，并且表达更高水平的PD-1[14]。将CLL细胞与自体CD4^＋^ T细胞共培养后观察到CLL细胞存活率提高[15]。CLL的特征是获得性免疫系统失调，T细胞发生多种功能障碍无法发挥抗肿瘤作用[16]，肿瘤细胞的免疫逃逸机制导致自体NK细胞无法消除CLL细胞。但是CLL患者的外周NK细胞不仅数量较正常人增加，而且在脱颗粒、细胞因子产生和抗体依赖的细胞介导的细胞毒作用方面具有功能性[17]。通过足够的刺激保留或恢复NK细胞功能可以作为新的免疫治疗策略，例如构建CAR-NK细胞。

（二）TIL与淋巴瘤

淋巴瘤是人体免疫系统的恶性肿瘤，免疫原性很强。临床上应用抗体及CAR-T细胞治疗取得了较好的疗效。目前，国内外不断地有研究学者尝试应用TIL治疗。

弥漫性大B细胞淋巴瘤（DLBCL）是最常见的B细胞非霍奇金淋巴瘤。已有研究能够证明靶向TME的联合疗法是有效的。Li等[18]利用小鼠B细胞淋巴瘤细胞系A20诱导的小鼠模型验证了评估秦皇方剂（QHF）联合阿霉素（ADM）的抗肿瘤作用，并在全身药理学的指导下探讨了其潜在机制：QHF是通过增加TME中的TIL来提高ADM的抗淋巴瘤作用。Apollonio等[19]通过研究人和小鼠的DLBCL细胞，确定了异常重塑的成纤维细胞网状细胞（FRC）网络的存在。在进行功能测定时，发现DLBCL中在FRC中表达改变的趋化因子和黏附分子导致了TIL迁移能力下降，而且以抗原特异性方式抑制CD8^+^ TIL细胞毒性。进一步的实验证明了靶向抑制FRC可以恢复TIL功能。为了克服免疫逃逸，治疗方案的优化可以通过抑制FRC来增加抗淋巴瘤TIL细胞毒性。郑州大学的高全立等[20]应用TIL治疗3例复发难治B细胞淋巴瘤，报告结果显示，复发难治淋巴瘤患者的TIL易于扩增。对患者化疗预处理后应用TIL治疗的有效率高，不良反应小。

套细胞淋巴瘤（MCL）细胞经历代谢和（或）功能衰竭，会抑制效应T细胞功能，损害抗肿瘤免疫力，导致MCL患者接受CAR-T治疗后复发。Yao等[21]在T细胞培养物中的定制代谢调节增强了TME中的TIL代谢适应性和抗肿瘤免疫力，最终可能会加强T细胞疗效。

LAG-3作为抗癌免疫治疗的一个靶点，可以与多种配体相互作用，通过抑制T细胞增殖活化来调节其功能。Moerdler等[22]的研究旨在表征LAG-3在儿童霍奇金淋巴瘤（HL）中的表达模式和临床意义。该研究发现73/115（63％）患者的LAG-3染色呈阳性，并且表达密度与患者的无事件生存率呈正相关。这一研究对于LAG-3作为儿童HL的免疫靶点方面具有创新性。

原发性皮肤B细胞淋巴瘤，腿型（PCLBCL-LT）是最具侵袭性的PCBCL。TME在其发展中起着至关重要的作用。Menguy等[23]在一个23例患者的独立队列中证明了增殖性TIL（CD3^+^Ki-67^+^细胞）在PCLBCL-LT中的数量明显高于惰性PCBCL（*P*＝0.0036），患者无进展生存（PFS）期也随之延长（*P*＝0.022）。这一发现表明，增殖性TIL可能作为PCLBCL-LT的有利预后因素，其刺激可能对治疗产生积极影响。未来会有更多的研究尝试应用TIL治疗各类淋巴瘤，为淋巴瘤的治疗和预后判断提供新策略。

（三）TIL与多发性骨髓瘤（MM）

在MM患者中，ALC作为宿主免疫状态的替代标志物，对患者具有预后意义，诊断时的ALC与新诊断的MM患者的生存有关[24]。Narwani等[25]对38例接受环磷酰胺、沙利度胺和地塞米松治疗的未经选择的MM患者进行了回顾性分析，发现治疗29 d后ALC水平较高的MM患者OS期延长，这提示患者自身免疫在MM控制中发挥着重要作用。

MM的TME特征为几种炎症趋化因子（CXCR3受体配体CXCL9和CXCL10）的表达水平上调，通过干扰CXCR4功能来限制NK细胞在骨髓中的定位。Bonanni等[26]研究显示CXCR3靶向促进骨髓的NK细胞积累对于IL-15活化NK细胞的持久抗骨髓瘤反应至关重要。相较于IL-2活化的NK细胞，IL-15活化的NK细胞在体内抑制MM生长的能力更持久。因此，利用IL-15激活NK细胞并靶向CXCR3可能有助于增强当前的MM治疗策略的疗效。MM患者通常伴有免疫失调，机体对肿瘤细胞的免疫反应与治疗和预后密切相关。Alrasheed等[27]对MM患者骨髓中的T细胞进行了检测，发现与正常人相比，患者骨髓中的Treg细胞数量更多，PFS期较短，并且具有明显的Treg免疫检查点特征（PD-1、LAG-3增加）。这些免疫参数在未来的研究中可能会协助预测MM患者的生存，并为免疫治疗提供潜在的靶点。

（四）TIL与其他恶性血液系统疾病

骨髓增生异常综合征（MDS）被认为是一种癌前病变，其核心特征是髓系细胞分化及发育异常，导致血细胞减少、造血功能衰竭和自身免疫失调，同时具有高风险向AML发展的倾向。根据Mailloux等[28]对FOXP3^+^ Treg细胞的研究表明，其数量和（或）激活状态都可能会影响MDS患者的肿瘤进展。

儿科MDS最常见的是儿童难治性血细胞减少症（RCC）。Aalbers等[29]通过流式细胞术分析了患者外周血中的T细胞亚群。结果显示CD4^+^/CD8^+^ T细胞比率紊乱、幼稚CD8^+^ T细胞减少，效应CD8^+^ T细胞和活化的CD8^+^ T细胞增多。这些发现为RCC患者的免疫抑制治疗提供了重要的实验依据。

三、TIL临床应用的挑战

当前限制TIL疗法广泛应用的一个主要问题是如何高效提取并培养TME的TIL并扩增至临床治疗所需数量，在扩增过程往往会因为受到培养环境微小的变化就导致细胞分化或表型发生变化[30]。TIL在体内的持久性对于恶性血液系统疾病来说较为重要[31]。目前在TIL与黑色素瘤的研究中，Rosenberg等[32]发现对患者进行非骨髓性淋巴细胞清除后再回输TIL治疗可以显著增强TIL的增殖能力和持久性。具体结果显示：93例患者中有52例（56％）患者有客观应答，20例（22％）患者达到完全缓解。这给增强TIL应用于血液肿瘤治疗的持久性带来了新的启发。另一个值得关注的问题是TME中的代谢限制和可溶性因子会加剧TIL细胞的功能衰竭，导致肿瘤细胞发生免疫逃逸，继续生长和转移[33]。尽管已开发的免疫检查点阻断抗体在临床试验中显示出不错的效果，但对于许多肿瘤，存在对免疫检查点阻断抗体的原有或获得性耐药性，增加了复发的风险。此外，为维持TIL细胞活性和细胞毒性而使用的IL-2在人体内常会导致细胞因子风暴、毛细血管泄漏综合征[34]、免疫抑制等不良反应[35]。

TIL实际应用到临床中还存在着许多问题，需要不断进行实验与探索，优化扩增方法、增强持久性、减少不良反应都是未来研究发展的趋势。

四、TIL的应用前景

相较于淋巴因子激活杀伤细胞（LAK）细胞，TIL被认为更适合作为细胞过继免疫治疗的效应细胞[8]。首先，TIL对肿瘤细胞的杀伤活性和特异性更强，LAK细胞对于晚期肿瘤无效时，TIL能够发挥出治疗效果；TIL在体外扩增输注患者体内后对IL-2依赖性低，不良反应发生率也降低，同时也可减轻患者的经济负担。其次，配合其他化疗药物和免疫调节因子开展以TIL为主的综合免疫治疗，可以进一步提高免疫治疗的疗效。TIL中肿瘤反应性T细胞的缺乏、耗尽和终末分化状态会导致治疗效果不够理想。最近，Islam等[36]将耗尽的TIL经过重新编程，使之携带具有针对多种肿瘤抗原反应性的T细胞受体（TCR）的重组诱导多功能干细胞，从而恢复其活力并获得更有效的免疫治疗效果。这种方法不仅恢复了TIL的活力，而且保留了对肿瘤特异性TCR的特性，对于血液肿瘤的未来治疗展示了巨大的潜力。

五、结语

TIL作为一种高效的免疫效应细胞，在肿瘤治疗领域展现出了巨大的潜力。它们拥有对抗肿瘤细胞的强大杀伤能力，并通过增强肿瘤患者的免疫系统，有效地抑制肿瘤的生长。特别是在恶性血液系统疾病的治疗中，TIL的研究开辟了新的视角和策略，其在肿瘤微环境中的作用不仅反映了免疫系统与肿瘤之间的复杂相互作用，而且还展示了其在预测疗效、评估预后及作为治疗靶点的巨大潜力。未来，通过基因工程技术改造TIL、开发新的免疫调节因子以及联合其他免疫治疗方法，有望提高TIL治疗的安全性和效果。TIL的进一步研究和应用将推动肿瘤免疫治疗领域的发展，为患者提供更有效的治疗方案。
